# Supplementary material for: Rapid and sensitive detection of genome contamination at scale with FCS-GX
Source: Genome Biol. 2024 Feb 26;25:60. doi: 10.1186/s13059-024-03198-7 (PMC10898089; doi:10.1186/s13059-024-03198-7)
Supplement: Supplementary file 2 — Additional file 2. Supplementary figures. [file 13059_2024_3198_MOESM2_ESM.pdf]

Supplementary material for

Rapid and sensitive detection of genome contamination at scale with FCS-GX

Alexander Astashyn, Eric S. Tvedte, Deacon Sweeney, Victor Sapojnikov, Nathan Bouk, Victor Joukov, Eyal Mozes, Pooja K. Strope, Pape M. Sylla, Lukas Wagner, Shelby L. Bidwell, Larissa C. Brown, Karen Clark, Emily W. Davis, Brian Smith-White, Wratko Hlavina, Kim D. Pruitt, Valerie A. Schneider, Terence D. Murphy

Additional file 1: supplementary figures

Fig. S1

Summary of FCS-GX results for false negatives in sensitivity tests. For 1 kbp sequence sets, aggregate counts of false negatives are shown for FCS-GX runs while including the same species taxids as the source genome during the alignment stage (+species) and while excluding same species taxids (-species). Categories are classified as follows: Review – sequences with the FCS-GX action REVIEW that are assigned the proper contaminant taxonomy but with subthreshold alignment coverage, Virus – sequences assigned prokaryote virus in prokaryote genomes and eukaryote virus in eukaryote genomes, Contaminant (inter-kingdom) – sequences assigned as contaminant by FCS-GX but the taxonomic classification is wrong and is in a different kingdom grouping. Contaminant (intra-kingdom) – sequences assigned as contaminant by FCS-GX but the taxonomic classification is wrong and is the same kingdom grouping. Non-contaminant – sequences assigned as non-contaminant. See **Additional file 2: Table S3** for counts/percentages of all false negative categories.

Fig. S2

Plots of aggregate FCS-GX alignment coverage against sensitivity. Aggregate coverage is calculated as the total percentage of the genome with overlaps from sequences in the FCS-GX reference database.

Results are shown for 1 kbp sequence sets for FCS-GX runs while including the same species taxids as the source genome during the alignment stage (blue circles) and while excluding same species taxids (red triangles).

Fig. S3

Complete distributions of specificity measurements. Distributions are shown for artificially fragmented genomes in six "kingdom" groups. Specificity is shown for genomes fragmented at three different window sizes (1 kbp, 10 kbp, 100 kbp). For each window size, specificity is shown for FCS-GX runs while including the same species taxids as the source genome during the alignment stage (+species) and while excluding same species taxids (-species). Red arrows point to ten outliers that are not visualized in **Fig. 2B**.

Fig. S4

Sensitivity and specificity measurements on non-mixed versus mixed sequence sets from two organisms. The sensitivity and specificity values for non-mixed sets are those calculated for 1 kbp and 100 kbp fragments, respectively. The sensitivity and specificity values for each mixed set represent values calculated for five experimental replicates (in cases where contaminant 1 kbp fragments exceed 5% length of host 100 kbp fragments) or one experimental replicate (if the contaminants are less than or equal to 5%). Sensitivity and specificity is shown for runs conducted with the host and contaminant species taxids included (+ species) and excluded (- species) from alignments.

Fig. S5

Sensitivity and specificity measurements on non-mixed versus chimeric mixed sequence sets from two organisms. The taxonomic scope is restricted to inter-kingdom host-contaminant pairs. The sensitivity and specificity values for non-mixed sets are those calculated for 1 kbp and 100 kbp fragments,

respectively. The sensitivity and specificity values for each chimeric mixed set represent values calculated for five experimental replicates (in cases where contaminant 1 kbp sequence counts exceed host 100 kbp sequence counts) or one experimental replicate (if contaminant sequence counts are less than or equal to host sequence counts). Sensitivity and specificity is shown for runs conducted with the host and contaminant species taxids included (+ species) and excluded (- species) from alignments.

Fig. S6

Length distribution of contaminants detected by FCS-GX.

Fig. S7

FCS-GX commands and sample output. The sample FCS-GX contamination action report displays 20/321 contaminated sequences identified in GCA_000006565.2.

Fig. S1

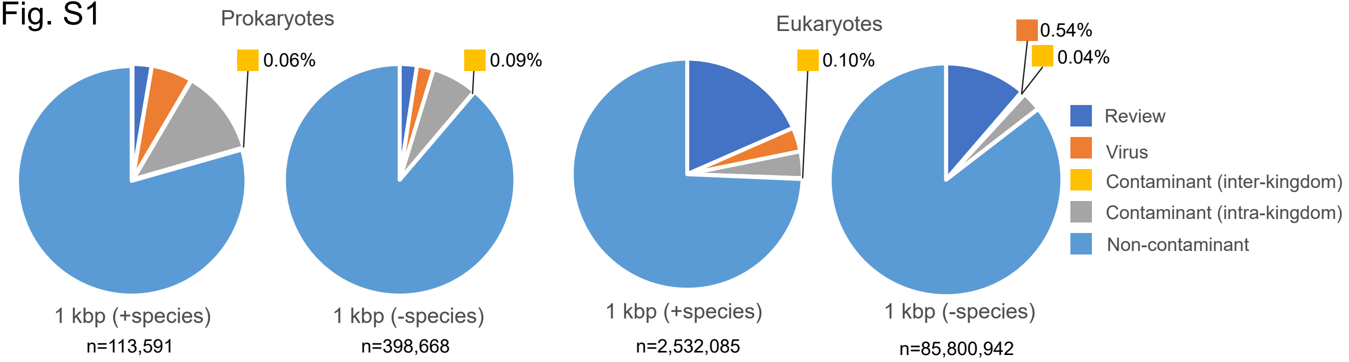

Prokaryotes
0.06%
0.09%
1 kbp (+species)
n=113,591
1 kbp (-species)
n=398,668
Eukaryotes
0.10%
0.54%
0.04%
1 kbp (+species)
n=2,532,085
1 kbp (-species)
n=85,800,942
Review
Virus
Contaminant (inter-kingdom)
Contaminant (intra-kingdom)
Non-contaminant

Fig. S2

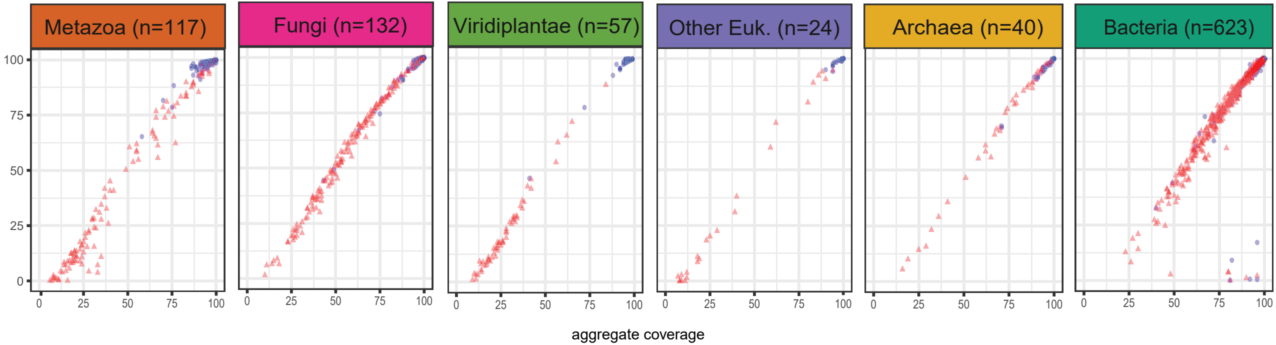

Metazoa (n=117)
Fungi (n=132)
Viridiplantae (n=57)
Other Euk. (n=24)
Archaea (n=40)
Bacteria (n=623)
0
25
50
75
100
aggregate coverage

Fig. S3

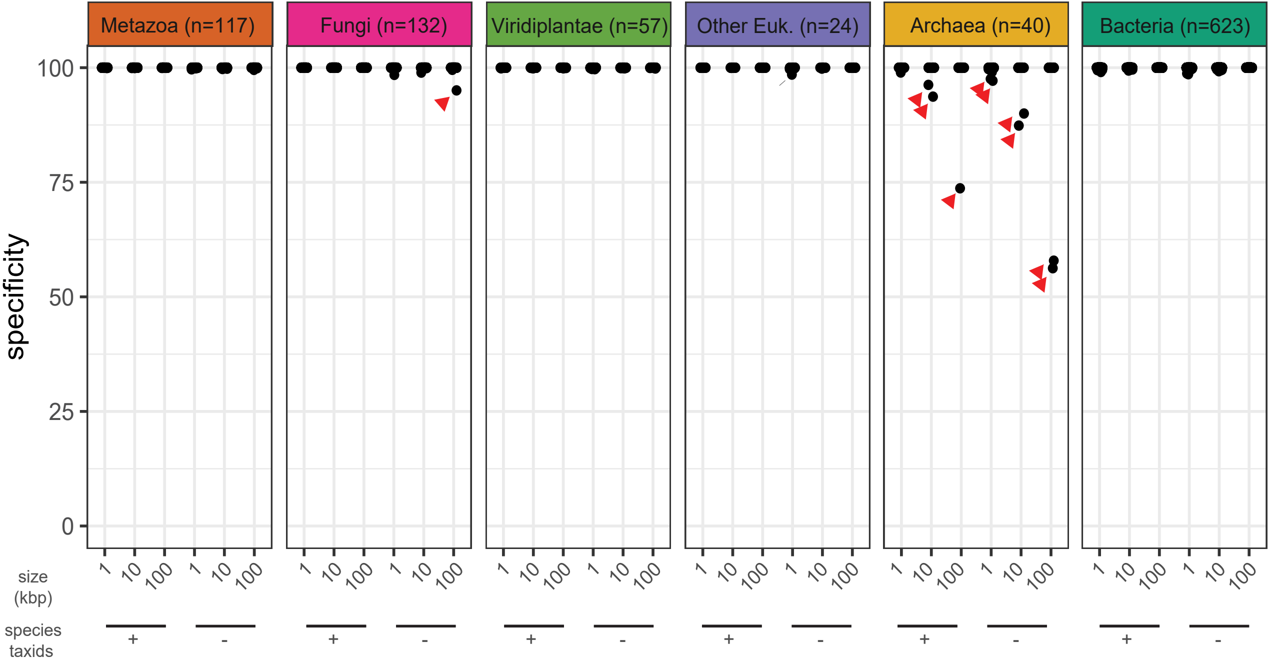

Metazoa (n=117)
Fungi (n=132)
Viridiplantae (n=57)
Other Euk. (n=24)
Archaea (n=40)
Bacteria (n=623)
specificity
100
75
50
25
0
size (kbp)
1
10
100
species taxids
+
-

Fig. S4

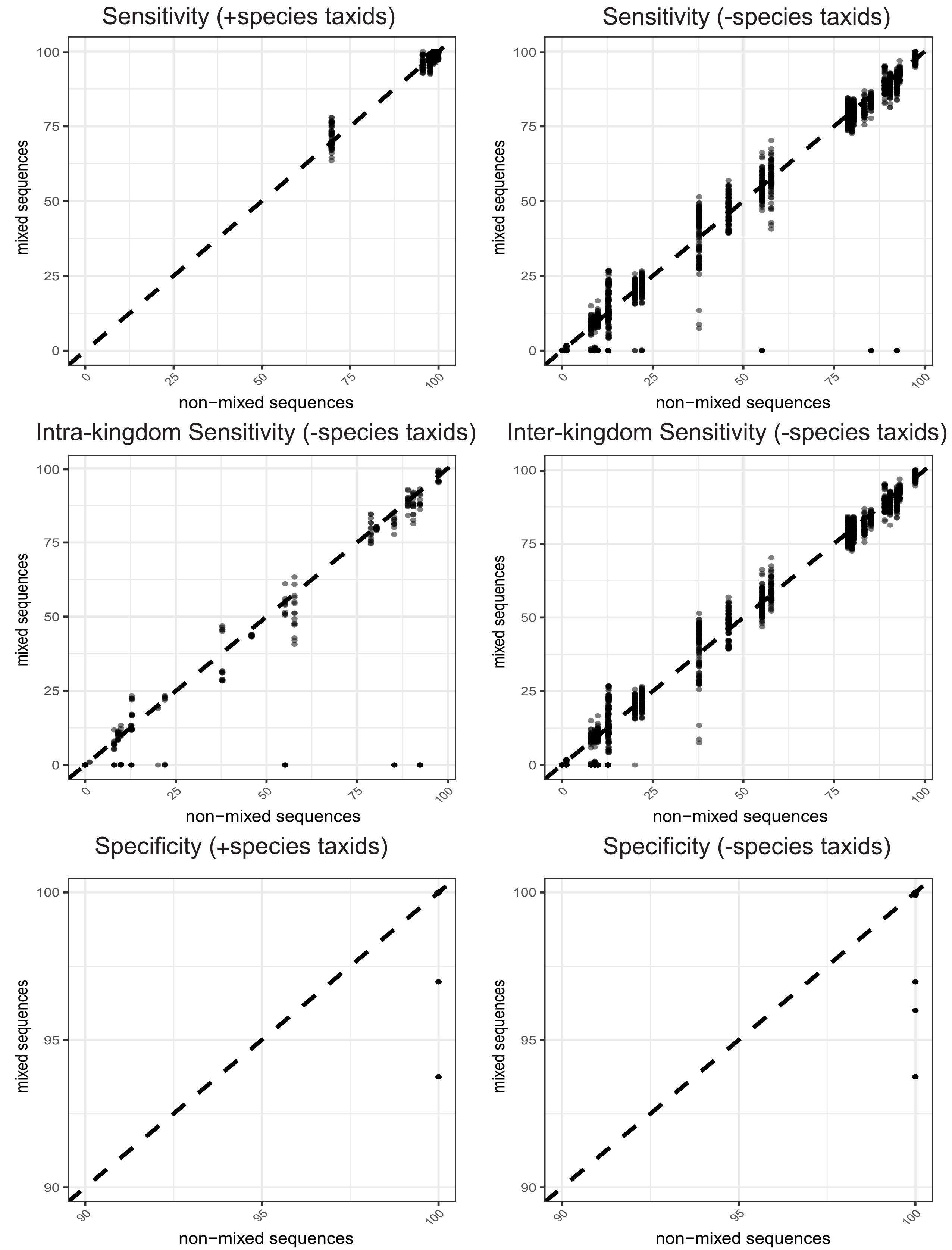

Sensitivity (+species taxids)
Sensitivity (-species taxids)
Intra-kingdom Sensitivity (-species taxids)
Inter-kingdom Sensitivity (-species taxids)
Specificity (+species taxids)
Specificity (-species taxids)
mixed sequences
non−mixed sequences
0
25
50
75
100
90
95

Fig. S5

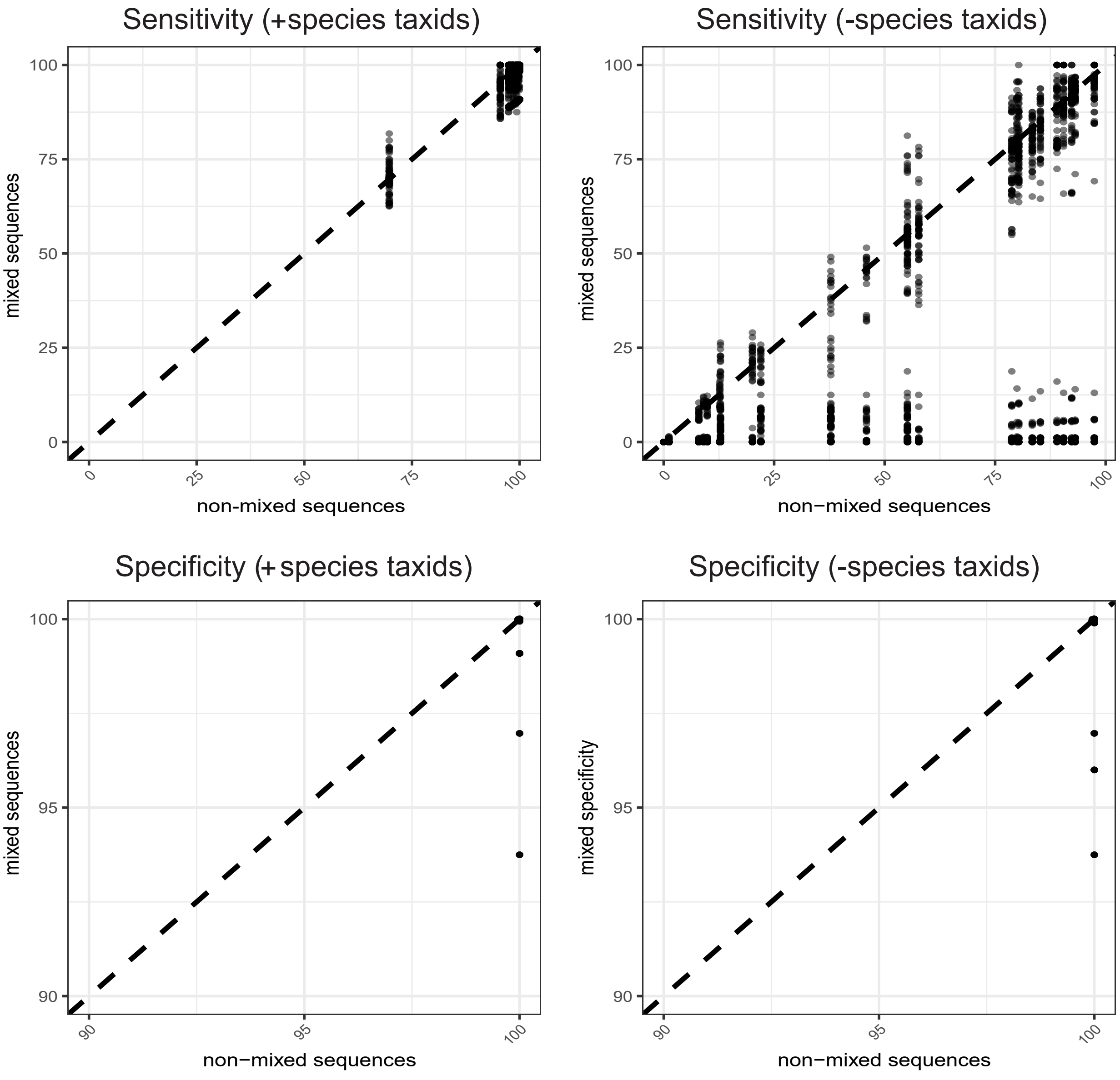
Sensitivity (+species taxids)
mixed sequences
non-mixed sequences
0
25
50
75
100
Sensitivity (-species taxids)
mixed sequences
non−mixed sequences
Specificity (+species taxids)
mixed sequences
non−mixed sequences
90
95
100
Specificity (-species taxids)
mixed specificity
non−mixed sequences

Fig. S6

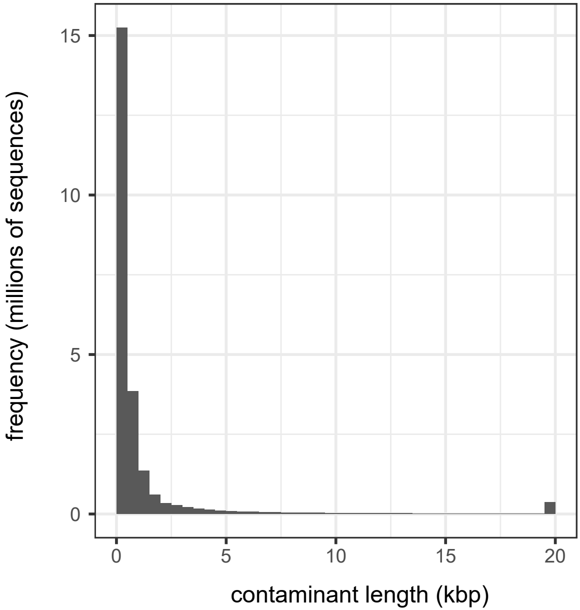

15
10
5
0
frequency (millions of sequences)
0
5
10
15
20
contaminant length (kbp)

FCS-GX command for Toxoplasma gondii ME49 (GCA_000006565.2)

```
python3 ./fcs.py screen genome --fasta ./gx_in/GCA_000006565.2_TGA4_genomic.fna.gz
--out-dir ./gx_out/ --gx-db "$GXDB_LOC/gxdb" --tax-id 508771
```

Summary contamination report for Toxoplasma gondii ME49 (GCA_000006565.2)

```
fcs_gx_report.txt contamination summary:
----------------------------------------
                                 seqs      bases
                                ----- ----------
TOTAL                             321     226690
-----                           ----- ----------
anml:primates                     282     169672
anml:nematodes                     34      55025
virs:eukaryotic viruses             3        819
anml:rodents                        1        639
fung:basidiomycetes                 1        535

------------------------------------------------------------------------------

fcs_gx_report.txt action summary:
---------------------------------
                                 seqs      bases
                                ----- ----------
TOTAL                             321     226690
-----                           ----- ----------
EXCLUDE                           321     226690
```

Sample FCS-GX contamination action report for Toxoplasma gondii ME49 (only a subset of rows shown)

#seq_id	start_pos	end_pos	seq_len	action	div	agg_cont_cov	top_tax_name
KE139043.1	1	1975	1975	EXCLUDE	anml:nematodes	100	Brugia malayi
KE139071.1	1	1494	1494	EXCLUDE	anml:primates	100	Homo sapiens
KE139077.1	1	1328	1328	EXCLUDE	anml:nematodes	81	Brugia malayi
KE139078.1	1	1466	1466	EXCLUDE	anml:nematodes	100	Brugia malayi
KE139079.1	1	1557	1557	EXCLUDE	anml:nematodes	100	Brugia malayi
KE139081.1	1	1439	1439	EXCLUDE	anml:nematodes	97	Brugia malayi
KE139286.1	1	1949	1949	EXCLUDE	anml:primates	100	Homo sapiens
KE139334.1	1	1575	1575	EXCLUDE	anml:nematodes	100	Brugia malayi
KE139857.1	1	503	503	EXCLUDE	anml:primates	96	Homo sapiens
KE139858.1	1	433	433	EXCLUDE	anml:primates	100	Homo sapiens
KE139863.1	1	395	395	EXCLUDE	anml:primates	100	Homo sapiens
KE139864.1	1	335	335	EXCLUDE	anml:primates	100	Homo sapiens
KE139866.1	1	634	634	EXCLUDE	anml:primates	100	Homo sapiens
KE139867.1	1	558	558	EXCLUDE	anml:primates	100	Homo sapiens
KE139870.1	1	519	519	EXCLUDE	anml:primates	100	Macaca fascicularis
KE140786.1	1	313	313	EXCLUDE	virs:eukaryotic viruses	74	Macaca fascicularis
KE140910.1	1	639	639	EXCLUDE	anml:rodents	97	Perognathus longimembris
KE140963.1	1	535	535	EXCLUDE	fung:basidiomycetes	100	Amanita rubescens

FCS-GX command for removing contaminant sequences (genome cleaning)

```
zcat GCA_000006565.2_TGA4_genomic.fna.gz | python3 ./fcs.py clean genome
--action-report ./gx_out/GCA_000006565.2_TGA4_genomic.fna.508771.6973.fcs_gx_report.txt
--output clean.fasta --contam-fasta-out contam.fasta
```
