# Supplementary material for: Rapid and sensitive detection of genome contamination at scale with FCS-GX
Source: Genome Biol. 2024 Feb 26;25:60. doi: 10.1186/s13059-024-03198-7 (PMC10898089; doi:10.1186/s13059-024-03198-7)
Supplement: Supplementary file 3 — Additional file 3. Review history. [file 13059_2024_3198_MOESM3_ESM.docx]

**1^st^ round**

**Reviewer 1**

Astashyn and Tvedte et al. address the critical issue of genomic contamination in genomic assemblies, which are foundational for scientific studies. The paper introduces FCS-GX, a tool that detects contamination by aligning assemblies to taxonomically labelled reference genomes in the FCS-GX database using a seed-and-extend approach. Notably, FCS-GX capability to detect intra-family contaminations is a novel feature not present in previous contamination detection tools. The authors applied their method to the NCBI database, specifically GenBank and RefSeq, to identify previously known and new contaminants. In summary, the proposed method is important and highly needed. By implementing it with the NCBI it is clear that the method will be needed and the analysis of GenBank and Refseq already created a valuable resource.

My main concerns are mainly benchmarking and providing details about the method.

Major

- The authors currently use megaBLAST as the time reference for comparing long DNA alignments, which is not considered state-of-the-art. As a reader, I would expect a comparison with minimap2 or another long-read mapper.

- The paper would benefit from a more detailed workflow figure for FCS-GX database creation and alignment process. As well as the exact criteria applied to remove something as contaminated. Currently, Figure 1 provides a high-level overview, which lacks granularity. Including a comprehensive workflow diagram with explicit steps would greatly help the understandability of the methodologies.

- In the benchmark presented, all contigs were contaminants. While real assemblies are a mixture of mostly correct contigs with contaminated ones, which might be separate contigs or even scaffolded into genomes. I recommended simulating a more realistic benchmark by incorporating contaminants into correct assemblies and capturing scaffolded in and separate contigs.

- The paper highlights the rapid speed of FCS-GX as one of its key features, but it falls short in explaining the algorithmic innovations or optimizations that contribute to this speed. For a methodological paper, as a reader, I anticipate sufficient details to potentially replicate or implement the methods discussed. However, the information provided in the manuscript is inadequate for such purposes. It is recommended that the authors include a more in-depth explanation of the algorithms and techniques that enable FCS-GX to achieve its notable speed, thereby enriching the paper's informational value and applicability.

- Contrary to other tools, FCS-GX is able to detect intra-kingdom contaminations, which is a great improvement over other methods. However, it is not clearly stated how this is done.

- The paper would benefit from clearly defining the thresholds used to label a contig as a contaminant and illustrating the trade-off between specificity and sensitivity based on these thresholds. Moreover, it would be insightful to measure specificity (Sp) and sensitivity (Sn) separately for contaminations across various taxonomic levels, such as inter-kingdom versus inter-family. This is important as detecting inter-kingdom contaminations might be inherently easier than inter-family ones, and distinguishing these metrics will offer a more nuanced understanding of the tool's performance.

- Please provide a list of identifiers of the 23,451,091 sequences and 2,932,319 annotated proteins.

Minor

- The selection of cross-kingdom contamination scans seems to be identical to Conterminators one. Please mention or cite it here: "FCS-GX exhibited high sensitivity across diverse samples from five tested "kingdom" groups (Metazoa, Viridiplantae, Fungi, other Eukaryotes, prokaryotes)", "We group taxonomic divisions into six broader kingdoms: animals (Metazoa), plants (Viridiplantae), fungi, protists (other Eukaryota), Bacteria, and Archaea".

- It is unclear what the consequence is if a contig is not aligned to any genome.

- Consistent upper-case: "(Metazoa, Viridiplantae, Fungi, other Eukaryotes, prokaryotes)" -> "(Metazoa, Viridiplantae, Fungi, other Eukaryotes, Prokaryotes) "

- In order to make the method more accessible please consider providing a conda package

Suggestion

- It is great that the contaminated sequences detected by FCS-GX will be labelled in the NCBI database. However, I think it would be fair to consider all tools that provided contaminated sequences when labelling sequences and not just FCS-GX. This would encourage the community to further try to detect contaminants and report them.

**Reviewer 2**

In their manuscript titled "Rapid and sensitive detection of genome contamination at scale with FCS-GX", Astashyn et al. (2023) introduce a novel method, FC-GX, for detecting contamination in assembled genomes at scale. The work addresses the critical need for an accurate method to detect and correct contaminated sequences. Contamination of publicly available sequencing data with misassembled sequences, environmental sequences, and technical artifacts presents numerous challenges and has time and again been implicated in erroneous conclusions. Early-stage detection and cleaning of data are crucial to ensuring high-quality scientific findings.

Throughout the manuscript, the authors use experiments with real assemblies and simulated conditions to demonstrate the high specificity of contamination detection, ensuring a low fraction of falsely flagged genomes. The method integrates several measures to warn of contamination, suggesting ways to clean sequences, allowing users to resolve problems not only in an automated way but also by manually resolving reported incongruencies.

Importantly, the method is designed not only for individual scientists but also to be integrated into large sequence repositories and databases. The efficiency and scalability of the method are crucial to improving the quality of the data used by millions of scientists daily.

The software installation was easy, with no unexpected behavior. Tested commands worked as expected, although not all commands could be tested due to the lack of the full-size database, which is provided but expectedly could not be utilized on a personal computer.

Overall, the manuscript presented by Astashyn et al. builds upon numerous previous works, improving them to allow for a more robust, accurate, and efficient screening of sequencing data for contaminants. This work has already been implemented as part of the largest sequence repository with numerous users and needs to be published. Despite the overall high quality of the manuscript and method, addressing several points could improve the quality of the work and ensure proper understanding by the readers.


Minor questions:

1. How is the hashed k-mer approach used in the manuscript different from the hashed k-mer databases used by other methods such as Kraken? Is dropping some of the positions, such as codon-wobble positions, the main difference? Have the authors compared a traditional k-mer hashmap to their h-mer implementation?

2. Since the purpose of the two-pass approach described in the Alignment section of the methods is to first identify candidate genomes and then refine the alignment in the second phase, what was the motivation for developing a custom implementation instead of using established tools for the first stage of the search? Also, if a secondary index is built on the fly as indicated by lines 493, then how important is the specificity of the first-stage search and can the database size be reduced via sketching such as kraken 2 implementation to allow faster and more memory-frugal implementation? I am certainly not asking for a re-implementation or change of the software, as the described method seems to be robust, rather I think it would be beneficial to include brief motivation for these choices.

3. The authors chose a representative set of genomes for testing, but no mention was made of viral genomes. Viral genomes are often present as common contaminants, and retroviral genomes, often sequenced as proviral sequence integrated into the host genome, can have host genome sequence misassembled upstream and downstream of the 5' and 3' LTRs. Multiple studies have reported misassembled HIV and SIV genomes, for instance. Considering the frequency with which viral genome assemblies are being generated, and that they are often used to detect viral presence in samples, contamination can also often lead to false findings. It would be helpful to include a note on viral genomes in the main body of the manuscript to guide users on whether they can expect correct behavior if they use the method on viral genomes. Specifically, is the FCS-GX suitable for contamination detection in viral genomes?

4. Following up on the viral genomes, have the authors checked for the presence of endogenous retro virus sequences in the contamination reports? Since those can be legitimate parts of assemblies, how would such cases be resolved?

5. Line 508-509: The authors mention limiting the number of reported contaminants per contaminated sequence. If a query sequence has multiple hits in the database (>2) could it signal that perhaps the region is homologous versus a contaminant?

6. Lines 431-433: the authors list several mechanisms they employ to create a representative database. However, it is not clear how many unique clean sequences remain unexplained by the database due to imposing 512 GB memory constraints on the total database size. Are there strategies that the authors could employ to create a more representative database? For instance, Kraken databases exist for larger and smaller sizes. Furthermore, recent additions to Kraken have facilitated the usage of large databases on machines with smaller RAM available. It seems that at the scale of NCBI, memory is likely not a bottleneck, while for individual contributors, there could be solutions to either provide smaller sets or load the database into memory in chunks. In the manuscript the authors explicitely state in comparison to the legacy implementation of a contamination screen that "CS-GX sensitivity increases are expected given its larger screening database". This suggests that not restricting the database might improve the accuracy even further. How significant is the reduction in accuracy due to not including more genomes in the database?

7. Any scripts used in the evaluation need to be made publicly available.

8. Is there a lower limit on the length of contaminated sequence that can be detected with the method, especially in cases where only a substring of the query is contaminated (due, for instance, to misassembly)? If so, mentioning of such limits in the manuscript could be very useful to the readers and users.

9. Supplementary Figure 1 presents a sample report for the T. gondii. Given the associations of the parasite with neurological disorders in humans, it is imperative that detection of the pathogen is reliable. As such, report of contamination of the T. gondii genome with human sequences (2 hits) was alarming. Is it expected that some of the reported sequences (as shown in the list below) are either highly repetitive or do not align with blast to the contaminant genome (human)?
- KE138932.1 - as a sanity check the first hit to the B. malayi seems valid based on both the protein and nucleotide sequence (megablast and blastp)
- KE139071.1 - hits human in accordance with the report, but the sequence is extremely repetitive.
- KE139286.1 - also very repetitive. Does not hit human with megablast or blastp (for the protein part contained in the sequence)

10: Lines 442-445: Did the authors check if any of the sequences in their database were implicated as potentially contaminated in other studies such as Conterminator?


Other questions and suggestions:

1. Supplemental figures are oddly labeled. While the names of the files provided by Genome Biology are correct, the names that appear when opening documents seem incorrect. For example, when opening supplemental figure 1 in firefox, the tab is labeled as Figure 5 which had me confused several times

2. Lines 127-137: I feel like these sentences could be paraphrased to make it clearer which genome, human or alphaproteobacteria, was available in the database and in the query for which experiment.

3. Figure S1. At the time the figure is referenced in the manuscript, no mentioning is made of the various output statuses of FCS-GX software, so the reader is unaware of what "Review" and "subthreshold alignment coverage" mean. Perhaps the caption could be made to explain it a bit better.

4. Line 237: I assume that "half of overall contamination" refers to the number of basepairs rather than the number of genomes or scaffolds. This needs to be clarified.

5. Line 257-258. Are the 233 genomes all of the remaining cases with >=90% contaminated sequence? If so, the wording in the sentence could be improved to make it clearer. If not, what are the rest?

6. Lines 449-458:. Perhaps separating this paragraph under a new sub-heading would be fitting? While the entirety of the section discusses database construction, this particular sub-section seems more technical and could warrant its own subheading.

Thank you

**Authors’ response**

Reviewer #1: Astashyn and Tvedte et al. address the critical issue of genomic contamination in genomic assemblies, which are foundational for scientific studies. The paper introduces FCS-GX, a tool that detects contamination by aligning assemblies to taxonomically labelled reference genomes in the FCS-GX database using a seed-and- extend approach. Notably, FCS-GX capability to detect intra-family contaminations is a novel feature not present in previous contamination detection tools. The authors applied their method to the NCBI database, specifically GenBank and RefSeq, to identify previously known and new contaminants. In summary, the proposed method is important and highly needed. By implementing it with the NCBI it is clear that the method will be needed and the analysis of GenBank and Refseq already created a valuable resource.

My main concerns are mainly benchmarking and providing details about the method.

Major

1. The authors currently use megaBLAST as the time reference for comparing long DNA alignments, which is not considered state-of-the-art. As a reader, I would expect a comparison with minimap2 or another long-read mapper.

RESPONSE: While newer aligners are available, megaBLAST is still quite commonly used for contamination analyses in part because of the ready availability of various BLAST databases from NCBI and some features of BLAST databases such as embedded taxonomy information. In addition to its 15 year history of use here at NCBI for contamination screening, parts of the legacy NCBI FCS pipeline have been reproduced by various large scale submitters such as JGI and the Broad Institute for contamination screening prior to submission. megaBLAST is also used by BlobToolKit. The FCS-GX versus megaBLAST compute time comparison included in the paper (lines 217-219) is provided to give readers a sense of the improved speed of genome contamination screening across a very wide scope of genome sequences (all submissions over a six month period). Developing a comparable screening approach based on another aligner such as minimap2 would be a completely new project and well outside the scope of this paper. Furthermore, minimap2 is designed to conduct pairwise alignments between genome assemblies of closely related species (less than 15% divergence) whereas FCS-GX conducts alignments between a query genome and a large reference database with analysis of multiple alignments and not just the top hits.

2. The paper would benefit from a more detailed workflow figure for FCS-GX database creation and alignment process. As well as the exact criteria applied to remove something as contaminated. Currently, Figure 1 provides a high-level overview, which lacks granularity. Including a comprehensive workflow diagram with explicit steps would greatly help the understandability of the methodologies.

RESPONSE: The intention of Figure 1 is to give readers a general idea of the FCS-GX workflow steps from input assembly to cleaned assembly in particular aimed at potential users of the software (our primary target audience for the paper); brief descriptions of steps are included to aid understanding. There are complex components of the workflow that would be challenging to illustrate and interpret visually, e.g. the comprehensive accounting of alignment post-processing used to categorize sequences into host vs. contaminant bins. The reviewer comment directed our attention to the information provided in Figure 1 regarding FCS-GX database creation, specifically that a reader might assume from the figure that the database contains the entire RefSeq+GenBank collection. We added text to the figure stating that database creation includes an assembly selection step as well as a capped RAM footprint. The Figure 1 legend states that FCS-GX uses a custom database (lines 972- 973), and the Methods section provides additional details to the summaries present in Figure 1 regarding database construction and alignment. We agree with the reviewer for the need for additional details on the criteria to assign contaminants, but we believe this is more appropriately addressed in the text (lines 604-674; see response to Reviewer 1, comment 6).

3. In the benchmark presented, all contigs were contaminants. While real assemblies are a mixture of mostly correct contigs with contaminated ones, which might be separate contigs or even scaffolded into genomes. I recommended simulating a more realistic benchmark by incorporating contaminants into correct assemblies and capturing scaffolded in and separate contigs.

RESPONSE: We ran two additional experiments to address this reviewer comment.

First, we ran FCS-GX on mixtures of contigs from two organisms: the full 100 kbp sequence set from one organism was treated as the host, and addition of up to 5% of 1 kbp fragments from a second organism was treated as the contaminant. We tested both intra- and inter- kingdom host-contaminant pairs. We added detailed methods for this experiment (lines 718-729). The distribution of sensitivity measurements for mixed sequence sets was largely consistent with sensitivity measurements on non-mixed 1 kbp sequences (lines 194-197; Additional file 2, Figure S4), thus sensitivity calculations on non-mixed sequences are appropriate approximations of the values expected in realistic scenarios. We discussed rare scenarios where experimental sensitivity was zero.

Second, we ran FCS-GX on chimeric mixtures of contigs from two organisms by adding 1 kbp contaminant fragments to the ends of 100 kbp sequences separated by 10 ‘N’ bases since the most common assembly artifacts involve mis-joins with assembly gaps. We measured sensitivity as the frequency of calling the appropriate TRIM on the short chimeric region. We added detailed methods for this experiment (lines 729-735). This test did reveal some decrease in sensitivity due to poor alignment coverage at chimera boundaries, particularly when same-species tax-ids were excluded from alignment steps (lines 206-212; Additional file 2, Figure S5). We prioritized specificity in the design of FCS-GX contamination calling, and importantly there was a low incidence of false positives in these two new experiments. Since discerning between true positive chimeras and false positive chimeras can be challenging, particularly in cases where alignment coverage on the host species is low, we err on underreporting these as contaminants. This is one area for future improvement.

4. The paper highlights the rapid speed of FCS-GX as one of its key features, but it falls short in explaining the algorithmic innovations or optimizations that contribute to this speed. For a methodological paper, as a reader, I anticipate sufficient details to potentially replicate or implement the methods discussed. However, the information provided in the manuscript is inadequate for such purposes. It is recommended that the authors include a more in-depth explanation of the algorithms and techniques that enable FCS-GX to achieve its notable speed, thereby enriching the paper's informational value and applicability.

RESPONSE: Our contamination screening design required an aligner that can efficiently align whole genomes to a large and diverse library of genomes comprising the Tree-of-Life, with the focus on common contaminants. Although the FCS-GX aligner does not use any fundamentally novel theoretical algorithms under the hood, it is engineered from the ground-up to take advantage of the multi-core, high-memory hardware that is readily available in both local and cloud environments. Additional details of the implementation have been expanded (lines 520-538). A summary of the optimizations include the splitting of query sequences into 100 kbp chunks for parallel processing, splitting h-mers for sorting arrays and conducting searches for matches, and memory mapping of the index file.

5. Contrary to other tools, FCS-GX is able to detect intra-kingdom contaminations, which is a great improvement over other methods. However, it is not clearly stated how this is done.

RESPONSE: We have re-organized and added text to the Results section (lines 121- 125) to make clear that FCS-GX uses both divisions of “kingdom” groups and divisions of BLAST names to assign sequences, enabling intra-kingdom contamination detection. We also added to the ‘Taxonomic assignment’ subsection of the Methods (lines 591-593) to re-iterate the use of BLAST name groupings as the basis for FCS- GX divisions.

6. The paper would benefit from clearly defining the thresholds used to label a contig as a contaminant and illustrating the trade-off between specificity and sensitivity based on these thresholds. Moreover, it would be insightful to measure specificity (Sp) and sensitivity (Sn) separately for contaminations across various taxonomic levels, such as inter-kingdom versus inter-family. This is important as detecting inter-kingdom contaminations might be inherently easier than inter-family ones, and distinguishing these metrics will offer a more nuanced understanding of the tool's performance.

RESPONSE: Because the aim of FCS-GX is not just to generate taxonomic labels for sequences but to also remove contaminant sequences that have discordant taxonomic assignments relative to the user-supplied tax-id, there is no universal threshold used to make a contamination call. Multiple factors for contamination assignment include the alignment identity-based score and coverage, whether the hits are intra-kingdom or inter-kingdom, whether the hit is to a division that is assigned as a high-confidence contaminant for the assembly based on the aggregate statistics (i.e. other sequences having top-hits to same putative contaminant tax-div), whether the labeling of sequences in NCBI databases necessitates special treatment (e.g. viruses), whether the contaminant spans the entire sequence or is a chimera, and whether the chimera case is likely endosymbiont.

We provide additional details regarding the motivation of logic design choices and provide specific values of thresholds for calling contaminants in various scenarios (lines 603-672). We also cite the future exploration of thresholding solutions to minimize intra-kingdom false positives, as the reviewer points out is a particularly difficult challenge (line 415-418).

Regarding separately measuring inter- vs intra-kingdom sensitivity and specificity, we already commented in the text that reduced sensitivity is primarily “due to poor alignment coverage or inconclusive taxonomic assignments resulting in non- contaminant calls and not due to incorrect taxonomy assignments” (lines 159-161), and specificity values are already very high leaving little margin for improvement by considering only inter-kingdom false positives. Consequently, there wouldn’t be much benefit to reporting Sn or Sp based only on inter-kingdom differences.

7. Please provide a list of identifiers of the 23,451,091 sequences and 2,932,319 annotated proteins.

RESPONSE: The sequence identifiers are available on the NCBI FTP site at https://ftp.ncbi.nlm.nih.gov/genomes/TOOLS/FCS/reports/current/publication_data/. The directory URL is presented in the ‘Availability of data and materials’ section (lines 796-797). The sequence identifiers are in the gx_details* files, and proteins in the gx_proteins* files.

Minor

8. The selection of cross-kingdom contamination scans seems to be identical to Conterminators one. Please mention or cite it here: "FCS-GX exhibited high sensitivity across diverse samples from five tested "kingdom" groups (Metazoa, Viridiplantae, Fungi, other Eukaryotes, prokaryotes)", "We group taxonomic divisions into six broader kingdoms: animals (Metazoa), plants (Viridiplantae), fungi, protists (other Eukaryota), Bacteria, and Archaea".

RESPONSE: The defined groups for cross-kingdom contamination is similar to Conterminator, but not identical, and derived from groups used in NCBI resources for many years. We clarified that FCS-GX uses eight "kingdom" groups to classify sequences (animals, fungi, plants, protists, bacteria, archaea, virus, synthetic)(lines 121-123). For example, FCS-GX can detect bacteria contamination in an archaeal genome (different than Conterminator). We now plot archaea results separately in Figure 2 and Figure 3 to reflect this distinction. Moreover, the Conterminator paper itself cites a different paper (Federhen 2012 Nucleic Acids Research; https://doi.org/10.1093/nar/gkr1178) when discussing the definition of "kingdom" taxonomic groupings, and a second paper published around the same time as the Conterminator paper (Schoch et al. Database 2020; https://doi.org/10.1093/database/baaa062) also includes information about kingdom groups defined by NCBI taxonomy. Thus it would appear that these are more appropriate sources to cite in this context (now citations 21,22). We do acknowledge Conterminator's important contribution to detection of contaminated entries in NCBI databases (lines 77-79) as well as provide a high-level comparison of contaminated sequences detected by Conterminator versus FCS-GX (lines 321-337).

9. It is unclear what the consequence is if a contig is not aligned to any genome.

RESPONSE: We added a sentence stating input sequences with zero alignments are assigned as inconclusive and not included in the final report, which is focused on listing definitive contaminants (line 628).

10. Consistent upper-case: "(Metazoa, Viridiplantae, Fungi, other Eukaryotes, prokaryotes)" -> "(Metazoa, Viridiplantae, Fungi, other Eukaryotes, Prokaryotes) "

RESPONSE: Thank you for pointing out this consistency issue. As a consequence of the response to question 8 from Reviewer 1, we separated visualizations for Bacteria and Archaea in Figures 2 and 3 since FCS-GX can detect cross-kingdom contamination in prokaryotes. We use upper case when referring to the scientific "kingdom" group names (e.g. Metazoa) and lower case when referring to common names (e.g. animals) or in combined "kingdom" groups (eukaryotes, prokaryotes). Lower case "eukaryotes" is distinguished between the "kingdom" group "other Eukaryotes" when necessary.

11. In order to make the method more accessible please consider providing a conda package.

RESPONSE: FCS-GX is now available as a conda package; the accessibility of the package is now listed in the “Availability of data and materials” section (lines 798-799).

Suggestion

12. It is great that the contaminated sequences detected by FCS-GX will be labelled in the NCBI database. However, I think it would be fair to consider all tools that provided contaminated sequences when labelling sequences and not just FCS-GX. This would encourage the community to further try to detect contaminants and report them.

RESPONSE: We agree that users may benefit from additional analyses; however, it is beyond the focus of this paper and involves considerable technical challenges. For example, published datasets from Conterminator are from 2018-2019, representing a small fraction of the assemblies available today, and not organized by assemblies which would complicate reporting. Data from BlobToolKit is also only available for a small fraction of assemblies and does not explicitly label contaminants, instead requiring manual review and analysis. NCBI does now routinely run CheckM and ANI on all prokaryote assemblies and publicly exposes the results, but the exponential growth of submission data requires that we limit the analyses that we provide to those that are computationally scalable.

Reviewer #2: In their manuscript titled "Rapid and sensitive detection of genome contamination at scale with FCS-GX", Astashyn et al. (2023) introduce a novel method, FC-GX, for detecting contamination in assembled genomes at scale. The work addresses the critical need for an accurate method to detect and correct contaminated sequences. Contamination of publicly available sequencing data with misassembled sequences, environmental sequences, and technical artifacts presents numerous challenges and has time and again been implicated in erroneous conclusions. Early- stage detection and cleaning of data are crucial to ensuring high-quality scientific findings.

Throughout the manuscript, the authors use experiments with real assemblies and simulated conditions to demonstrate the high specificity of contamination detection, ensuring a low fraction of falsely flagged genomes. The method integrates several measures to warn of contamination, suggesting ways to clean sequences, allowing users to resolve problems not only in an automated way but also by manually resolving reported incongruencies.

Importantly, the method is designed not only for individual scientists but also to be integrated into large sequence repositories and databases. The efficiency and scalability of the method are crucial to improving the quality of the data used by millions of scientists daily.

The software installation was easy, with no unexpected behavior. Tested commands worked as expected, although not all commands could be tested due to the lack of the full-size database, which is provided but expectedly could not be utilized on a personal computer.

Overall, the manuscript presented by Astashyn et al. builds upon numerous previous works, improving them to allow for a more robust, accurate, and efficient screening of sequencing data for contaminants. This work has already been implemented as part of the largest sequence repository with numerous users and needs to be published. Despite the overall high quality of the manuscript and method, addressing several points could improve the quality of the work and ensure proper understanding by the readers.

Minor questions:

1. How is the hashed k-mer approach used in the manuscript different from the hashed k-mer databases used by other methods such as Kraken? Is dropping some of the positions, such as codon-wobble positions, the main difference? Have the authors compared a traditional k-mer hashmap to their h-mer implementation?

RESPONSE: The primary difference is dropping codon-wobble positions and using a reduced 1-bit {AG, CT} alphabet. The intent is to increase sensitivity in coding regions. We have added statements (lines 113-116; lines 517-519) and Supplementary Table S1 to compare k-mers vs h-mers in a small test set of genomes.

2. Since the purpose of the two-pass approach described in the Alignment section of the methods is to first identify candidate genomes and then refine the alignment in the second phase, what was the motivation for developing a custom implementation instead of using established tools for the first stage of the search? Also, if a secondary index is built on the fly as indicated by lines 493, then how important is the specificity of the first-stage search and can the database size be reduced via sketching such as kraken 2 implementation to allow faster and more memory-frugal implementation? I am certainly not asking for a re-implementation or change of the software, as the described method seems to be robust, rather I think it would be beneficial to include brief motivation for these choices.

RESPONSE: The first alignment pass generates the initial weak alignments to be refined, with the scope of refinement limited to the most promising taxa. Kraken can report that query sequences and subsequences correspond to specific taxon but does not produce information about the corresponding locations on reference sequences. The size of the database is reduced by sampling the positions in the reference with a fixed-sized stride (10 bp stride for prokaryotes and 20 bp stride for eukaryotes; lines 514-515), which reduces the size of the database index by approximately the same factor.

We have added to the Methods section to clearly state that the first pass generates alignments (i.e. is not just a candidate genome identification mechanism as the Reviewer mentions) and that the second pass refines alignments (lines 557-559).

3. The authors chose a representative set of genomes for testing, but no mention was made of viral genomes. Viral genomes are often present as common contaminants, and retroviral genomes, often sequenced as proviral sequence integrated into the host genome, can have host genome sequence misassembled upstream and downstream of the 5' and 3' LTRs. Multiple studies have reported misassembled HIV and SIV genomes, for instance. Considering the frequency with which viral genome assemblies are being generated, and that they are often used to detect viral presence in samples, contamination can also often lead to false findings. It would be helpful to include a note on viral genomes in the main body of the manuscript to guide users on whether they can expect correct behavior if they use the method on viral genomes. Specifically, is the FCS-GX suitable for contamination detection in viral genomes?

RESPONSE: We thank the reviewer for pointing out this missing detail in the text. FCS-GX can assign sequences as viruses, and this detail has been added to the Results section (lines 121-123). Non-viral contaminants and virus sequences from a discordant superkingdom can be identified (lines 640-642). However, we did not perform large-scale testing on viral genomes in NCBI databases and as such we are reluctant to say that FCS-GX will produce satisfactory genome cleaning results on viral genomes. We now mention this as an area for future investigation (lines 423-424).

4. Following up on the viral genomes, have the authors checked for the presence of endogenous retro virus sequences in the contamination reports? Since those can be legitimate parts of assemblies, how would such cases be resolved?

RESPONSE: Genomes containing known endogenous viral elements were included in tests when developing FCS-GX contamination assignment logic. To prevent erroneous labeling of endogenous viral elements as contaminants, FCS-GX currently only removes eukaryotic virus sequences from eukaryote assemblies if they are identified as non-chimeric with insufficient evidence for being integrated with host sequence (i.e. FCS-GX action “EXCLUDE”). Eukaryote sequences with evidence of both virus and host, which are likely to represent a biological integration event, as well as any prokaryote virus sequence in prokaryote genomes, are currently ignored by contamination cleaning processes to avoid false reports of biological retrovirus integration. This is now detailed (lines 642-644).

5. Line 508-509: The authors mention limiting the number of reported contaminants per contaminated sequence. If a query sequence has multiple hits in the database (>2) could it signal that perhaps the region is homologous versus a contaminant?

RESPONSE: The reviewer is correct in suggesting that multiple database hits could be the consequence of shared homology, and we have elaborated on these details in the methods. There are multiple safeguards to minimize the false positives occurring in homologous regions. First, we calculate aggregate statistics of alignments for each division, and divisions with at least 75% alignment overlap with the asserted host division or at least 75% alignment overlap with regions calculated as highly conserved are treated as primary divisions (i.e. host sequences) (lines 617-623). Second, in inter- kingdom eukaryote matches, individual sequences with signal >50% conserved sequence content are ignored for contamination calling (lines 601-626 for conserved definition, lines 626-628 for threshold).

6. Lines 431-433: the authors list several mechanisms they employ to create a representative database. However, it is not clear how many unique clean sequences remain unexplained by the database due to imposing 512 GB memory constraints on the total database size. Are there strategies that the authors could employ to create a more representative database? For instance, Kraken databases exist for larger and smaller sizes. Furthermore, recent additions to Kraken have facilitated the usage of large databases on machines with smaller RAM available. It seems that at the scale of NCBI, memory is likely not a bottleneck, while for individual contributors, there could be solutions to either provide smaller sets or load the database into memory in chunks. In the manuscript the authors explicitly state in comparison to the legacy implementation of a contamination screen that "FCS-GX sensitivity increases are expected given its larger screening database". This suggests that not restricting the database might improve the accuracy even further. How significant is the reduction in accuracy due to not including more genomes in the database?

RESPONSE: From the early design stages, we decided that being able to run FCS-GX on a commodity hardware is not a requirement, allowing for engineering choices that take advantage of modern powerful hardware that is available in some local compute farms and is ubiquitously available on major cloud platforms. The FCS-GX distribution is cloud-ready, making it accessible for any entity, from a grad student to a major sequencing center. However, it is highly beneficial for genome producers to screen themselves, which led to targeting a 512 GiB server as a reasonable compromise available to many high-volume submitters.

Although it is possible in theory to shard the database, align the query genome against multiple shards on small-memory hosts(s), and combine the resulting alignments for the downstream processing, it has serious downsides. First, it complicates the pipeline design. Second, it deviates from the self-imposed requirement that external users should be able to run the same pipeline that is performed internally at NCBI. Third, it transforms the time complexity of the algorithm to become linear in the size of the reference database, which would rapidly negate the inherent speed advantages in the algorithm. In particular, the various bulk analyses of the initial h-mer hits and alignments would add substantial overhead if performed in shards with subsequent integration.

We don’t have statistics on the number of unexplained sequences. We have emphasized coverage in the database of organisms most likely to be environmental contaminants, including diverse prokaryotes, fungi, and protists, which is reflected in the high sensitivity measurements for these taxonomic groups. An important component of ongoing work for FCS-GX is the evaluation of database contents, but a full rebuild and comprehensive assessment of accuracy with different database sizes would be a considerable undertaking and is outside the scope of this manuscript. In measuring accuracy, any additions that increase sensitivity would have to be weighed against the possibility of introducing additional contaminated entries that could produce false positives.

7. Any scripts used in the evaluation need to be made publicly available.

RESPONSE: Scripts used in the analysis and visualization of data are available on the NCBI FTP site at

https://ftp.ncbi.nih.gov/genomes/TOOLS/FCS/reports/20230416/publication_scripts/

(lines 797-798).

8. Is there a lower limit on the length of contaminated sequence that can be detected with the method, especially in cases where only a substring of the query is contaminated (due, for instance, to misassembly)? If so, mentioning of such limits in the manuscript could be very useful to the readers and users.

RESPONSE: The GX aligner can find hits as small as 60 bp during the first alignment pass (lines 607-608). However, the “lower limit on the length of contaminated sequence that can be detected” is different from the “lower limit on the length of contaminated sequence that can be reported.” For the latter point, there are multiple factors that determine contaminant assignment (see response to Reviewer 1, question 6). The least stringent reporting criteria for a contaminant sequence span is an alignment score of 50 or inter-kingdom span with 80% alignment coverage (lines 624- 625), which is sufficient to identify contaminant sequences as small as 200 bp (the INSDC minimum size) with a moderately similar match in the database (the score is dependent on sequence composition so a score of 50 does not correspond to a specific percent identity). Specifically in cases of chimeras, currently only inter-kingdom chimeras are designated for cleanup. Sequence spans less than 10 kbp assigned as intra-kingdom chimeras are ignored (lines 634-635), and spans greater than 10 kbp are marked as GX action REVIEW (manual investigation recommended, but not auto- cleaned)(lines 685-687).

9. Supplementary Figure 1 presents a sample report for the T. gondii. Given the associations of the parasite with neurological disorders in humans, it is imperative that detection of the pathogen is reliable. As such, report of contamination of the T. gondii genome with human sequences (2 hits) was alarming. Is it expected that some of the reported sequences (as shown in the list below) are either highly repetitive or do not align with blast to the contaminant genome (human)?

- KE138932.1 - as a sanity check the first hit to the B. malayi seems valid based on both the protein and nucleotide sequence (megablast and blastp)

- KE139071.1 - hits human in accordance with the report, but the sequence is extremely repetitive.

- KE139286.1 - also very repetitive. Does not hit human with megablast or blastp (for the protein part contained in the sequence)

The presence of human contamination in this Toxoplasma genome has been documented (Lu & Salzberg 2018; https://doi.org/10.1371/journal.pcbi.1006277). FCS- GX is able to identify repetitive sequences as contaminants so long as there is sufficient evidence for the same source of contamination in non-repetitive regions. This is crucial since repetitive sequences from contaminating organisms can be easier to assemble due to its elevated copy number. KE139286.1 appears to have tandem repeats of the pentamer ATTCC found in HSATII. If the reviewer ran a megablast search on KE139286.1 while filtering out low-complexity or human species-specific repeats, then the absence of search results is unsurprising.

10: Lines 442-445: Did the authors check if any of the sequences in their database were implicated as potentially contaminated in other studies such as Conterminator?

RESPONSE: We have used an iterative process to remove contaminant sequences from the FCS-GX database, as well as excluding small sequences that are more likely to be contaminants but contribute little to the screening process. FCS-GX also leverages alignments to multiple taxonomic divisions as a way to mitigate the effects of residual contamination in the database. We’ve also included a way to quickly exclude hits to sequences in the database identified as suspect (lines 502-504). Cross-referencing the results from Conterminator vs the database contents found 1,433 suspect sequences (0.05% of total database sequences). 410 (28%) of the sequences appear to be true positives confirmed by FCS-GX most of which have been added to the current exclusion list after review. Conversely, at least 608 (42%) are likely false positives based on the reported Conterminator hit itself being identified as a contaminant by FCS-GX (e.g., a likely directionality problem similar to the mouse example described at lines 325-329), which confounds leveraging the data for further database cleanup without deeper review for a later time. We decided not to include these details because the issues with false positives over-emphasize potential issues in the Conterminator dataset, which we’ve already mentioned in brief and is not particularly relevant to our paper.

Other questions and suggestions:

11. Supplemental figures are oddly labeled. While the names of the files provided by Genome Biology are correct, the names that appear when opening documents seem incorrect. For example, when opening supplemental figure 1 in firefox, the tab is labeled as Figure 5 which had me confused several times

RESPONSE: Thank you for pointing this out! Apparently the PDF files had the incorrect title property that was preserved when the files were renamed. These have been fixed in both the main and supplemental figures.

12. Lines 127-137: I feel like these sentences could be paraphrased to make it clearer which genome, human or alphaproteobacteria, was available in the database and in the query for which experiment.

RESPONSE: We added additional details to the Results section (lines 145-147) to provide a specific example of a genome we tested and how the sensitivity experiment was run. By stating explicitly that the tested genomes and “mock hosts” are in the database, this should help readers better understand the set of experiments simulating novel organisms when same-species alignments are excluded (lines 162-165).

13. Figure S1. At the time the figure is referenced in the manuscript, no mentioning is made of the various output statuses of FCS-GX software, so the reader is unaware of what "Review" and "subthreshold alignment coverage" mean. Perhaps the caption could be made to explain it a bit better.

RESPONSE: We removed the call to Figure S1 in the Results section (line 136). The statement that FCS-GX identifies and removes contamination with minimal user interaction doesn’t require Figure S1. Now, former Figure S1 (now Figure S7) is called in the Methods section when describing FCS-GX usage and the various contamination assignment labels (line 677).

14. Line 237: I assume that "half of overall contamination" refers to the number of basepairs rather than the number of genomes or scaffolds. This needs to be clarified.

RESPONSE: You are correct. Change made (line 282), thank you.

15. Line 257-258. Are the 233 genomes all of the remaining cases with >=90% contaminated sequence? If so, the wording in the sentence could be improved to make it clearer. If not, what are the rest?

RESPONSE: We identified inconsistencies with the reported numbers in this paragraph due to variation in calculations. The “1,040 genomes have extreme contamination by proportion of contaminated sequence” (lines 285-286) refers to GenBank genomes with contaminant fractions ≥90%, however the analysis used to identify subsets of taxonomic assignment issues on line 292-294 used a less stringent cutoff (≥60%), which wasn’t explained in the text. We changed the reporting and data in Table S11 to include only contaminant fractions ≥90%, and the genome count number and bp on line 279-280 reflects the remainder of genomes with contaminant fractions ≥90% that do not involve generic vs. specific taxonomic assignment issues.

16. Lines 449-458:. Perhaps separating this paragraph under a new sub-heading would be fitting? While the entirety of the section discusses database construction, this particular sub-section seems more technical and could warrant its own subheading. RESPONSE: We added the subheading “FCS-GX database structure” for this section (line 505).

**2^nd^ round**

**Reviewer 1**

I would like to thank the authors to address all my comments.

**Reviewer 2**

The revised manuscript by Astashyn et al. demonstrates significant improvements, effectively addressing most of the initial concerns. The text benefits from expanded methods, cleaner structure and improved reporting. Assuming the authors adequately address my remaining questions and other reviewers raise no additional major concerns, I recommend this manuscript for publication.

Comments:

1. I recommended that additional scripts be deposited for publication. Currently, only scripts used for parsing summaries and reproducing figures are available. Ideally, all scripts, including those involved in database generation, software execution, and summary table/file creation for figures, should be deposited alongside.

2. Consider relocating or duplicating the explanation of "BLAST name groupings" found in lines 121-125. Although the definition is introduced in these lines, the explanation is only provided in lines 591-593.

3. Can the authors provide brief explanation of how an alignment score is computed. Given its consistent use throughout the manuscript, and its distinction from percent identity, a dedicated explanation would enhance clarity for readers.

4. Regarding comparison of the database sequences with the Conterminator report. The authors list that 410 sequences are true positives and have been added to the exclusion list and 608 sequences are likely false positives. However these two sets together comprise only 70% of the 1,433 total overlapping sequences between Conterminator and the FCS-GX database. What are the remaining 415 sequences?

5. In Figure 1, there is confusion about the example in the illustration. It is unclear why, at the start, the cartoon depicts 7 lines, two of which (entirely or partially in red) are marked as contaminants. At the end of execution, 3 sequences are reported as contaminants, including one that was supposedly clean in the input. Additional clarification is necessary.

6. Regarding contamination of the T. gondii M49 genome sequence with human sequence. It is my understanding that Lu and Salzberg 2018 did not explicitly implicate human as the source of contamination in their report, rather noting presence of contamination in the M49 T. gondii assembly. Is my understanding of the workings of the software correct, that the KE139286.1 scaffold alone could not warrant assignment of human contamination on the basis of extremely low-complexity (due to being recognized as a repeat). If so, that leaves KE139071.1 as the scaffold used by the software to make assignment, which has higher complexity, albeit still repetitive. Perhaps it is worth mentioning this at least in the caption to the supplementary file? This is just a suggestion and not a recommendation.

Thank you

**Authors’ response**

Reviewer #1: I would like to thank the authors to address all my comments.

RESPONSE: We’d like to thank the reviewer again for their helpful comments.

Reviewer #2: The revised manuscript by Astashyn et al. demonstrates significant improvements, effectively addressing most of the initial concerns. The text benefits from expanded methods, cleaner structure and improved reporting. Assuming the authors adequately address my remaining questions and other reviewers raise no additional major concerns, I recommend this manuscript for publication.

Comments:

1. I recommended that additional scripts be deposited for publication. Currently, only scripts used for parsing summaries and reproducing figures are available. Ideally, all scripts, including those involved in database generation, software execution, and summary table/file creation for figures, should be deposited alongside.

RESPONSE: We have publicly available scripts for FCS-GX database generation available in the zenodo repository (fcs-gx-0.4.0/make_gxdb/). Scripts for software execution and summary table/file creation have been added alongside the fcs-gx-0.4.0 source code into a new zenodo repository so that nearly all the data/scripts used in the analysis for this paper are accessible from the same link. The exceptions are (1) the FCS-GX database files and (2) the FASTAs used in sensitivity/specificity experiments, as these data files exceed the 50 Gbp size requirement for zenodo. We have modified the ‘Availability of data and materials’ to reflect these changes.

2. Consider relocating or duplicating the explanation of "BLAST name groupings" found in lines 121-125. Although the definition is introduced in these lines, the explanation is only provided in lines 591-593.

RESPONSE: We have copied the definition to the results section.

3. Can the authors provide brief explanation of how an alignment score is computed. Given its consistent use throughout the manuscript, and its distinction from percent identity, a dedicated explanation would enhance clarity for readers.

RESPONSE: The definition is included as the last sentence of the end of the Methods

– FCS-GX alignment – Alignment section. We added the word “alignment” to make the definition clear to readers. It now reads “As such, we define the alignment score of a segment as the square root of the sum of squares of alignment lengths with 100% nucleotide identity.”

4. Regarding comparison of the database sequences with the Conterminator report. The authors list that 410 sequences are true positives and have been added to the exclusion list and 608 sequences are likely false positives. However these two sets together comprise only 70% of the 1,433 total overlapping sequences between Conterminator and the FCS-GX database. What are the remaining 415 sequences?

RESPONSE: These are sequences that were identified by Conterminator as putative contaminants, but were not readily classified as true positives or false positives on the basis of FCS-GX results. We will factor further analyses of these sequences into future efforts to detect and remove contaminated database sequences for a future database update.

5. In Figure 1, there is confusion about the example in the illustration. It is unclear why, at the start, the cartoon depicts 7 lines, two of which (entirely or partially in red) are marked as contaminants. At the end of execution, 3 sequences are reported as contaminants, including one that was supposedly clean in the input. Additional clarification is necessary.

RESPONSE: We have adjusted the figure to make the input/output of the cartoon example consistent, and added the following text to the figure legend: “In the cartoon example, one complete sequence and one partial sequence assigned as contaminant are removed from the input assembly to produce the final cleaned FASTA.”

6. Regarding contamination of the T. gondii M49 genome sequence with human sequence. It is my understanding that Lu and Salzberg 2018 did not explicitly implicate human as the source of contamination in their report, rather noting presence of contamination in the M49 T. gondii assembly. Is my understanding of the workings of the software correct, that the KE139286.1 scaffold alone could not warrant assignment of human contamination on the basis of extremely low-complexity (due to being recognized as a repeat). If so, that leaves KE139071.1 as the scaffold used by the software to make assignment, which has higher complexity, albeit still repetitive. Perhaps it is worth mentioning this at least in the caption to the supplementary file? This is just a suggestion and not a recommendation.

RESPONSE: In this case, human(=gx division primates) was identified as a high confidence source of contamination by the multiple criteria defined in the ‘Contamination assignment’ Methods section (we added details about this definition in the last round of revisions). As such, individual sequences corresponding to those divisions that are also repetitive can still be called contaminant. Figure S7 only includes a subset of action report rows, which isn’t fully representative of the contamination identified in the genome (e.g. there were 282 primate equences identified by FCS-GX but only two displayed in the Figure S7 report). The reviewer may have interpreted this to mean that KE139071.1 as the only other sequence shown in Figure S7 meant that sequence alone was responsible for the contamination call which is not correct. We changed the figure to include a greater diversity of rows reflecting the types of detected contamination and added clarification that only a subset of action report rows are displayed in the figure.
